# Rubisco catalytic properties of wild and domesticated relatives provide scope for improving wheat photosynthesis

**DOI:** 10.1093/jxb/erv574

**Published:** 2016-01-21

**Authors:** Anneke Prins, Douglas J. Orr, P. John Andralojc, Matthew P. Reynolds, Elizabete Carmo-Silva, Martin A. J. Parry

**Affiliations:** ^1^Plant Biology and Crop Science Department, Rothamsted Research, Harpenden AL5 2JQUK; ^2^International Maize and Wheat Improvement Center (CIMMYT), El Batán, Texcoco CP 56130,Mexico

**Keywords:** *Aegilops*, barley, carboxylation, enzyme kinetics, photosynthesis, Rubisco, Triticeae, *Triticum.*

## Abstract

Rubisco is a major target for improving crop photosynthesis and yield, yet natural diversity in catalytic properties of this enzyme is poorly understood. Rubisco from 25 genotypes of the Triticeae tribe, including wild relatives of bread wheat (*Triticum aestivum*), were surveyed to identify superior enzymes for improving photosynthesis in this crop. *In vitro* Rubisco carboxylation velocity (*V*_c_), Michaelis–Menten constants for CO_2_ (*K*_c_) and O_2_ (*K*_o_) and specificity factor (*S*_c/o_) were measured at 25 and 35 °C. *V*_c_ and *K*_c_ correlated positively, while *V*_c_ and *S*_c/o_ were inversely related. Rubisco large subunit genes (*rbc*L) were sequenced, and predicted corresponding amino acid differences analysed in relation to the corresponding catalytic properties. The effect of replacing native wheat Rubisco with counterparts from closely related species was analysed by modelling the response of photosynthesis to varying CO_2_ concentrations. The model predicted that two Rubisco enzymes would increase photosynthetic performance at 25 °C while only one of these also increased photosynthesis at 35 °C. Thus, under otherwise identical conditions, catalytic variation in the Rubiscos analysed is predicted to improve photosynthetic rates at physiological CO_2_ concentrations. Naturally occurring Rubiscos with superior properties amongst the Triticeae tribe can be exploited to improve wheat photosynthesis and crop productivity.

## Highlight

Catalytic characteristics of Rubisco from wild and domesticated wheat relatives show natural variation, with Rubisco from *Ae. cylindrica* and *H. vulgare* having the potential to improve photosynthesis in bread wheat.

## Introduction

Wheat is the most widely grown crop and an important source of protein and calories, providing more than 20% of the calories consumed worldwide (Braun *et al.*, 2010). It is projected that the world population will rise to over 9 billion by the year 2050 (United Nations, Department of Economic and Social Affairs, Population Division, 2013). This growth in population, along with a rise in *per capita* consumption (Kearney, 2010), will increase the global demand for food. Future increases in crop production will rely mainly on new strategies to increase yield and cropping intensity (Gregory and George, 2011; Alexandratos and Bruinsma, 2012; Fischer *et al.*, 2014).

Yield traits that were positively affected by the green revolution appear to have relatively little remaining potential for further exploitation in modern wheat (Zhu *et al.*, 2010), and further increases in yield potential will need to come from the improvement of photosynthetic efficiency. In this context, significant variation in biomass has been identified in exotic wheat genetic resources (Reynolds *et al.*, 2015). Rubisco, (EC.4.1.1.39) is a key player in photosynthetic CO_2_ assimilation, as it catalyses the first step of the Calvin–Benson cycle, fixing carbon dioxide through the carboxylation of ribulose-1,5-bisphosphate (RuBP). Rubisco also catalyses an additional and competing reaction with oxygen, which leads to the loss of fixed carbon and energy during photorespiration. This, together with the relatively low catalytic rate of Rubisco, limits photosynthetic productivity. Overcoming the limitations of Rubisco is therefore a major target in attempts to increase photosynthesis and yield (Parry *et al.*, 2007; Parry *et al.*, 2013).

There is natural variation in the catalytic properties of Rubisco isolated from various higher plants (Delgado *et al.*, 1995; Galmés *et al.*, 2005; Kapralov and Filatov, 2007; Andralojc *et al.*, 2014; Galmés *et al.*, 2014a; Galmés *et al.*, 2014b). Relatively few studies report all catalytic parameters—the maximum velocities (*V*) and the Michaelis–Menten constants (*K*_M_) for the carboxylase (c) and oxygenase (o) activities (*V*_c_, *V*_o_, *K*_c_, and *K*_o_, respectively) and the specificity factor (*S*_c/o_=(*V*_c_/*K*_c_)/(*V*_o_/*K*_o_))—or measurements at anything other than a single temperature. Greater natural diversity is likely to be revealed when the catalytic parameters of Rubisco from a broader range of species become available. Current evidence suggests that there is a trade-off between the maximum carboxylation rate of Rubisco (*V*_c_) and the relative specificity for CO_2_ (*S*_c/o_) (Bainbridge *et al.* 1995; Zhu *et al.*, 2004; Savir *et al.*, 2010), which may limit the extent to which these parameters can be independently altered. Clearly, a superior Rubisco for improving crop performance will have catalytic properties that maximize carboxylation, minimize the oxygenase activity, and enable enhanced rates of photosynthesis in relevant environments (Galmés *et al.*, 2014b; Sharwood and Whitney, 2014).

Evolution of Rubisco variants with differing catalytic properties has been driven by their respective diverse cellular environments, which in turn are affected by their respective external environments (see Carmo-Silva *et al.*, 2014 and references therein). These conditions provide selective pressures which favour changes in Rubisco structure that optimize performance (Tcherkez *et al.*, 2006). While the chloroplast-encoded Rubisco large subunits incorporate the catalytic sites and therefore contribute directly to the observed catalytic properties, recent evidence suggests that changes in expression of genes within the nuclear-encoded small subunit multigene family can also cause catalytic variation (e.g. Morita *et al.*, 2014).

The goal of this study was to identify Rubisco variants in the Triticeae tribe with catalytic properties that are likely to improve photosynthetic efficiency in wheat. We focused on wheat relatives so that useful traits could be introduced into a wheat genetic background by means of wide crossing (thus avoiding genetic manipulation), with increased likelihood that the available (wheat) chloroplast chaperones and Rubisco activase isoforms would subsequently promote the assembly and maintenance of catalytic activity in any resulting forms of the Rubisco holoenzyme. Triticeae genotypes from diverse climates and geographical locations were studied to increase the likelihood of identifying forms of Rubisco with different (and hopefully superior) kinetic properties from those found in *Triticum aestivum* (bread wheat). The resulting catalytic parameters were assessed *in silico* using a biochemical model of leaf photosynthesis. This approach suggested that Rubisco from two of the genotypes studied has the potential to improve the photosynthetic capacity and yield potential of wheat.

## Materials and methods

### Plant material and growth conditions

For all kinetic measurements, values obtained in test samples were compared with those of *T. aestivum* cv Cadenza, which was used as control. Cadenza is widely grown and has routinely been used in transformation experiments, making it a well-known and characterized variety. A total of 25 genotypes were analysed (Table 1). Species related to bread wheat were chosen with a range of characteristics, such as adaption to warmer conditions (*T. aestivum* SATYN and *T. dicoccon* CIMMYT), or which had been used to introduce desirable traits into bread wheat (Schneider *et al.*, 2008). Seeds were obtained from CIMMYT (Mexico); the Royal Botanic Gardens, Kew (UK); and colleagues at Rothamsted Research. All plants were grown from seed in trays containing Rothamsted Research compost mix in a glasshouse at 20 °C with a 16h photoperiod. Additional lighting was provided whenever the photosynthetically active radiation (PAR) fell below 500 μmol m^−2^ s^−1^. All plants were well watered. Young, healthy leaves were harvested 2–3 weeks after sowing and rapidly frozen in liquid nitrogen.

**Table 1.** *Triticeae genotypes used to survey Rubisco catalytic properties for improving photosynthesis of UK bread wheat* (T. aestivum *cv Cadenza*) Haploid genome according to Van Slageren (1994). First letter denotes chloroplast genome.

Identity	Species name	Other species name(s) and additional information	Haploid genome
*T. aestivum* (C)	*Triticum aestivum*	Spring wheat var. Cadenza	BA^u^D
*T. aestivum* SATYN1	*Triticum aestivum* SATYN_II_9410	PUB94.15.1.12/FRTL (CIMMYT line)	BA^u^D
*T. aestivum* SATYN2	*Triticum aestivum* SATYN_II_9440	WHEAR//2*PRL/2*PASTOR (CIMMYT line)	BA^u^D
*T. aestivum* SATYN3	*T. aestivum* SATYN_II_9428	MTRWA92.161/PRINIA/5/SERI*3//RL6010/4*YR/3/PASTOR/4/ BAV92 (CIMMYT line)	BA^u^D
*T. dicoccon*1	*Triticum dicoccon* CI12214	Emmer wheat; INTRID:CWI47369 ENT:2129 (CIMMYT line)	BA^u^
*T. dicoccon*2	*Triticum dicoccon* CI12214	Emmer wheat; INTRID:CWI47368 ENT:2128 (CIMMYT line)	BA^u^
*T. dicoccon*3	*Triticum dicoccon* PI355483	Emmer wheat; INTRID:CWI45495 ENT:255 (CIMMYT line)	BA^u^
*T. dicoccon*4	*Triticum dicoccon* CI12214	Emmer wheat; INTRID:CWI47366 ENT:2126 (CIMMYT line)	BA^u^
*T. timonovum*	*Triticum timonovum*	Synthetic octoploid of *T. timopheevii*	GA^m^
*T. timopheevii*	*Triticum timopheevii*	Sanduri wheat	GA^m^
*Triticale* (Talentro)	*Secale cereale* × *Triticum aestivum*	*× Triticosecale* cv Talentro; frost and drought tolerant	BA^u^R
*Triticale* (Rotego)	*Secale cereale* × *Triticum aestivum*	*× Triticosecale* cv Rotego; frost and drought tolerant	BA^u^R
*H. vulgare*	*Hordeum vulgare*	Barley var. Lenins; relatively drought tolerant, not cold tolerant	H
*Ae. tauschii*	*Aegilops tauschii*	*Aegilops squarrosa*; drought tolerant	D
*Ae. juvenalis*	*Aegilops juvenalis*		DMU
*Ae. vavilovii*	*Aegilops vavilovii*	Drought tolerant	DMS
*Ae. biuncialis*	*Aegilops biuncialis*	Drought tolerant	UM
*Ae. triuncialis*	*Aegilops triuncialis*	Barbed goatgrass; Millenium Seed Bank 47689; winter annual	UC
*Ae. comosa*	*Aegilops comosa*		M
*Ae. uniaristata*	*Aegilops uniaristata*		N
*S. cereale*	*Secale cereale*	Rye var. Agronom; frost and drought tolerant	R
*T. monococcum*	*Triticum monococcum*	Millenium Seed Bank 11008, einkorn	A^m^
*Ae. cylindrica*	*Aegilops cylindrica*	Jointed goatgrass; cold tolerant	DC
*Triticale* (Cando)	*Secale cereale* × *Triticum aestivum*	*× Triticosecale* cv Cando; frost and drought tolerant	BA^u^R
*Ae. speltoides*	*Aegilops speltoides*	Not frost tender	S
*B. distachyon*	*Brachypodium distachyon*	Purple false brome; Accession BD21; diploid inbred	

### Specificity factor

Rubisco from snap-frozen young leaves (at least 500cm^2^) was extracted in homogenization buffer (40mM triethanolamine pH 8.0, 10mM MgCl_2_, 0.5mM EDTA, 1mM KH_2_PO_4_, 1mM benzamidine, 5mM ε-aminocaproic acid, 50mM 2-mercaptoethanol, 5mM DTT, 10mM NaHCO_3_, 1mM phenylmethanesulfonyl fluoride (PMSF), 1% w/v insoluble polyvinylpolypyrrolidone (PVPP)) at 0.3ml cm^−2^. Leaves were homogenized in a pre-cooled blender for 45s and then filtered through four layers of muslin. Homogenate was clarified by centrifuging at 13 870×*g* for 12min at 4 °C, after which PEG_4000_ (60% w/v) was added to the supernatant to obtain a final concentration of 20.5% (w/v). MgCl_2_ was added to the solution to increase the concentration of MgCl_2_ to 20mM. The ensuing protein precipitation was complete after 30min at 0 °C, and the precipitate collected by centrifugation at 13 870×*g* for 20min at 4 °C. The protein precipitate was redissolved in column buffer containing 25mM TEA–HCl (pH 7.8), 5mM MgCl_2_, 0.5mM EDTA, 1mM ɛ-aminocaproic acid, 1mM benzamidine, 12.5% glycerol, 2mM DTT and 5mM NaHCO_3_. This was clarified by centrifugation at 175 000×*g* for 20min at 4 °C followed by filtration through a 0.45 µm regenerated cellulose syringe filter before further purification by anion-exchange chromatography on a 5ml HiTrap Q column (GE Healthcare, UK). Rubisco was eluted with a 0–1.0M linear NaCl gradient in the same buffer. Fractions with significant absorbance at 280nm were tested for Rubisco activity by measuring the RuBP-dependent incorporation of ^14^CO_2_ into acid-stable products, as detailed below. Fractions showing Rubisco activity were pooled and further purified by size-exclusion chromatography on a Sephacryl S-200 column (GE Healthcare, UK) using a buffer consisting of 50mM Bicine–NaOH, pH 8.0, 10mM MgCl_2_, 0.2mM EDTA, 10mM NaHCO_3_ and 2mM DTT. Peak fractions based on Rubisco activity were pooled and concentrated using Pierce Protein Concentrators (150K MWCO, Thermo Scientific, UK). Samples were snap-frozen in liquid nitrogen and stored at –80 °C. Before use, samples were desalted by gel filtration through Sephadex G50 (medium grade; Sigma-Aldrich, UK) pre-equilibrated with assay buffer (0.1M Bicine–NaOH, pH 8.2, 20mM MgCl_2_).

*S*_c/o_ was determined by measuring the decline in oxygen that accompanied the total consumption of RuBP in an oxygen electrode (Hansatech Instruments, UK), as described by Parry *et al.* (1989): Rubisco was activated in extracts by adding orthophosphate (4mM, pH 8.2) and NaHCO_3_ (11mM), and incubating at 37 °C for 40min. A reaction mixture containing assay buffer and carbonic anhydrase (0.001% w/v, ≥2500 W-A units mg protein^–1^; Sigma-Aldrich, UK) was equilibrated in an oxygen electrode vessel at controlled pH and temperature. All subsequent additions were made through a small aperture using glass syringes. Activated Rubisco and NaHCO_3_ (2mM) were added to the vessel and the oxygen signal allowed to stabilize. RuBP (0.37mM) was added to the reaction, which was allowed to run to completion over a few minutes, as indicated by a stabilized oxygen signal. The amount of RuBP carboxylated was calculated by subtracting the oxygenated amount (represented by the amount of oxygen consumed during the reaction) from the amount added. The specificity factor was calculated as follows:

$$\begin{array}{l} S_{\text{c}/\text{o}}=\left(\text{RuBP carboxylated}/\text{RuBP oxygenated}\right)\\ \;\times \;\left({\text{O}}_{\text{2}}\text{concentration}/{\text{CO}}_{\text{2}}\text{concentration}\right) \end{array}$$

RuBP was prepared as previously described (Wong *et al.*, 1980).

### Rubisco catalytic properties

Rubisco was extracted from 20–30cm^2^ of leaf material that was light-adapted immediately before being snap-frozen, then stored at –80 °C. Leaves were ground in an ice-cold mortar with 100mg quartz sand and 3.5ml of ice-cold extraction buffer, consisting of 100mM Bicine–NaOH, pH 7.9, 5mM MgCl_2_, 1mM EDTA, 2mM benzamidine, 5mM ε-aminocaproic acid, 10mM NaHCO_3_, 50mM 2-mercaptoethanol, 5% (w/v) PEG_4000_, 10mM DTT, 1% (v/v) plant protease inhibitor cocktail (Sigma-Aldrich, UK), 1mM PMSF and 2% (w/v) insoluble PVPP. After centrifugation for 5min at 14 000×*g* and 4 °C, samples were desalted by gel filtration through PD-10 columns (Sephadex G-25 Medium, GE Healthcare, UK) that had been pre-equilibrated with 100mM Bicine–NaOH, pH 8.0, 10mM MgCl_2_, 1mM EDTA, 1mM benzamidine, 1mM ε-aminocaproic acid, 1mM KH_2_P_i_, 10mM NaHCO_3_, 10mM DTT and 2% (w/v) PEG_4000_, 2% (v/v) protease inhibitor cocktail (Sigma-Aldrich, UK) and 20mM MgCl_2_ was added before samples were snap-frozen and stored in liquid nitrogen, awaiting assay.

Catalytic parameters were measured essentially as previously described (Carmo-Silva *et al.*, 2010). Carboxylation activity was measured at 8, 16, 24, 36, 68, and 100 µM CO_2_ (aq) in equilibrium with a gas phase of N_2_ supplemented with 0, 21, 60, or 100% (v/v) O_2_. *K*_M_ and *V*_max_ for carboxylation (*K*_c_ and *V*_c_, respectively) were calculated at each O_2_ concentration using a Michaelis–Menten kinetic model. *K*_M_ and *V*_max_ for oxygenation (*K*_o_ and *V*_o_, respectively) were calculated as follows:

$$K_{\text{o}}=\left[{\text{O}}_{\text{2}}\right]/\left[\left(K_{\text{M},\text{app}}/K_{\text{c}}\right)-\text{1}\right]$$

and

$$V_{\text{o}}=\left(V_{\text{c}}\times K_{\text{o}}\right)/\left(K_{\text{c}}\times S_{\text{c}/\text{o}}\right)$$

where *K*_c_ is the Michaelis–Menten constant for CO_2_ in the absence of O_2_, and *K*_M,app_ is the apparent Michaelis–Menten constant for CO_2_ as measured in the reactions equilibrated with 21, 60, or 100% O_2_. Specific mixtures of N_2_ and O_2_ were prepared using a gas divider (Signal Group, UK) and concentrations of O_2_ in solution were calculated at 100% relative humidity and standard atmospheric pressure (101.3 kPa). At 25 °C, the solubility of O_2_ was taken as 257.5 μM and the saturation vapour pressure of water as 11.6 kPa. At 35 °C, the solubility of O_2_ was taken as 216.6 μM and the saturation vapour pressure of water as 12.0 kPa (http://www.eidusa.com/Theory_DO.htm). The concentration of CO_2_ in solution (in equilibrium with HCO_3_^−^) was calculated assuming a p*K*_a_ of 6.11 at 25 °C and a p*K*_a_ of 6.06 at 35 °C for carbonic acid, taking into consideration the pH of each buffer solution (measured on the day of assay). Carbonic anhydrase (≥77 WA units per 1ml reaction; Sigma-Aldrich, UK) was present in the reaction solution to maintain equilibrium between NaHCO_3_ and CO_2_. Control reactions were performed by measuring CO_2_ fixation (acid-stable ^14^C) in reaction solutions lacking RuBP or NaHCO_3_, as well as following total inhibition of Rubisco by prior treatment with an excess of the tight-binding inhibitor 2-carboxyarabinitol-1,5-bisphosphate (CABP). These controls confirmed that the activity measured was entirely due to Rubisco.

Radioactive content of ^14^C-labelled compounds was measured in 0.4–0.45ml aqueous solutions to which were added 3.6ml Ultima Gold Scintillation cocktail (Perkin-Elmer, UK), in a Tri-Carb 2100TR Liquid Scintillation Analyser (Perkin-Elmer, USA).

Turnover number (*k*_cat_: mol product mol active site^−1^ s^−1^) was calculated from the corresponding *V*_max_ values (*V*_c_ and *V*_o_: µmol acid-stable ^14^C mg Rubisco^−1^ min^−1^).

### Rubisco quantification

Rubisco was quantified by the [^14^C]CABP binding assay described by Parry *et al.* (1997). For this, aliquots of the leaf extracts used in the assays described above, which had been snap-frozen immediately after extraction, were used. Each assay was performed in duplicate. Radioactive content of ^14^C-labelled compounds was measured as described above in ‘Rubisco catalytic properties’. Radiolabelled [2′-^14^C]CABP was prepared as previously described (Pierce *et al.*, 1980).

### Sequencing of Rubisco large subunit genes (*rbc*L)

Genomic DNA was extracted from young leaf tissue using the Qiagen DNEasy Plant Kit (Qiagen, UK). Partial *rbc*L fragments (equivalent to codons 1–463, *c.* 98% of the *rbc*L coding region) were amplified (Phusion HF polymerase, Invitrogen, USA) using the primers 5′rbcL_F2 (5′-TAATTCATGAGTTGTAGGGAGGG-3′) and cp063R (5′-TTTCCATACTTCACAAGCAGCAGCTAG-3′, from Dong *et al.*, 2013), and cloned using the pGEM T-Vector Easy System (Promega, UK) with blue-white selection. For each genotype, multiple colonies with the fragment incorporated were identified and sequenced using the Eurofins Genomics service (Eurofins Genomics EU, Germany). Sequencing was performed using the primers M13 rev (5′-CAGGAAACAGCTATGACC-3′), M13 uni (5′-TGTAAAACGACGGCCAGT-3′), DRS15 (5′-CAAAAGTAGTAGAAACCATTTTAGTTCAGGTGG-3′ and DRS19 (5′-GKGYTCCTATTGTAATGCATGACTACTTAAC-3′). Sequence data were analysed using Geneious 7 (Biomatters; Kearse *et al.*, 2012). Sequences obtained have been submitted to EMBL (http://www.ebi.ac.uk/ena/) and are publicly available (see Supplementary Table S1 at *JXB* online for accession numbers). Corresponding residue differences in the predicted large subunit (LSu) sequences appear in the format [Cadenza residue][residue position][test species residue] throughout the text.

### Photosynthesis modelling

The effect of replacing native Rubisco in a wheat leaf with Rubisco from another species was modelled at 25 and 35 °C by entering the measured Rubisco catalytic constants into the biochemical models of carboxylation-limited and RuBP-limited C_3_ photosynthesis (equations 2.20 and 2.23, respectively, in von Caemmerer (2000)). To accomplish this, values of *K*_c_, *K*_o_, and *S*_c/o_ were converted from units of concentration (mol l^−1^) to those of partial pressure (bar), assuming solubilities of 3.34×10^–2^ and 1.26×10^–3^ mol (l bar)^−1^ for CO_2_ and O_2_, respectively, for assays performed at 25 °C. At 35 °C, the respective solubilities were taken as 2.51×10^–2^ and 1.083×10^–3^ mol (l bar)^−1^. We assigned a value of 38 μmol m^−2^ for the estimated number of Rubisco active sites and kept this value constant for all samples. *R*_d_ was calculated as 0.015*V*_cmax_. We assumed *J*_max_ as 1.5*V*_cmax_ at 25 ºC and 35 ºC giving a good fit above *C*_a_. Equations used to generate the *A*–*C*_i_ curves were: 

$$A_{\text{c}}=<\left[\left(C_{\text{i}}–\Gamma *\right)V_{\text{cmax}}\right]/\left\{C_{\text{i}}+\left[K_{\text{c}}\left(\text{1}+{\text{O}}_{\text{2}}/K_{\text{o}}\right)\right]\right\}>–R_{\text{d}}$$

and 

$$A_{\text{j}}=\left\{\left[\left(C_{\text{i}}–\Gamma *\right)J_{\text{max}}\right]/\left(\text{4}C_{\text{i}}+\text{8}\Gamma *\right)\right\}–R_{\text{d}}$$

(von Caemmerer, 2000).

### Statistical methods

Best-fit values of Michaelis–Menten constants (*K*_c_ and *K*_o_) and maximum velocities (*V*_c_ and *V*_o_) were derived from the kinetic data using Sigmaplot (v12.5). There was one determination per test genotype, with Cadenza values calculated from *n*=7 for catalytic properties and *n*=9 for *S*_c/o_. Values of *S*_c/o_ at 25 °C were normalized to the corresponding value for the Rubisco of *T. aestivum* cv Cadenza (*S*_c/o_=100), which was determined in parallel to each test sample measured (Parry *et al.*, 1989). For *S*_c/o_, the mean±SEM for every Rubisco preparation was calculated from a minimum of five technical replicates. Correlation coefficients were calculated using the Pearson product moment correlation test. The interaction between genotype and temperature was analysed using a non-parametric statistical approach. Ranking was done in descending order with the highest rank assigned number 1. Ranks of the measured variables for each genotype at 25 °C and 35 °C were correlated using Spearman’s rank correlation coefficient and these were tested for statistical significance using Genstat (17th edn, VSN International Ltd, Hemel Hempstead, UK).

## Results

**Fig. 1.**
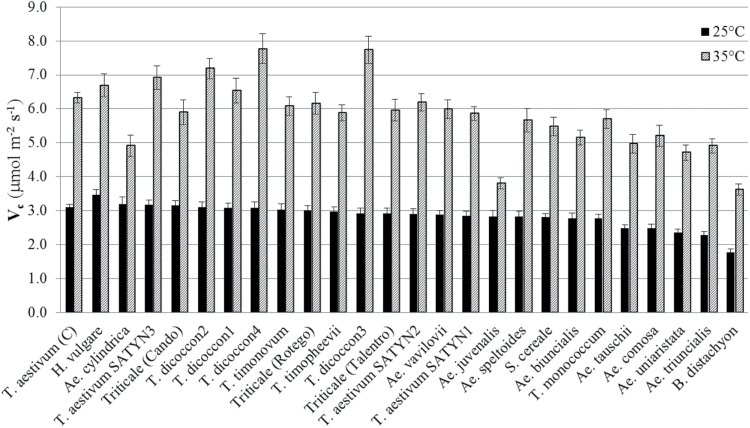
Rubisco carboxylation velocity (*V*_c_) at 25 °C (black bars) and 35 °C (hatched bars) in 25 Triticeae genotypes. Data organized in decreasing rank at 25 °C, except for *T. aestivum* cv Cadenza, which is shown on the far left-hand side for comparison.

Rubisco catalytic properties at 25 and 35 °C were determined for 25 genotypes of Triticeae. For all genotypes, the maximum carboxylation velocity (*V*_c_) was significantly higher at 35 °C than at 25 °C, ranging from 1.34 times higher in *Ae. juvenalis* to 2.65 times higher in *T. dicoccon3* (Fig. 1 and Supplementary Tables S2 and S3). At 25 °C, *H. vulgare* ranked the highest for *V*_c_, followed by *Ae. cylindrica*, *T. aestivum* SATYN3, and Triticale (Cando) above *T. aestivum* cv Cadenza (reference genotype). At 35 °C, *T. dicoccon*4 (a line developed by CIMMYT for warm climates) ranked the highest for *V*_c_, followed by the other CIMMYT lines (*T. aestivum* SATYN and *T. dicoccon* genotypes) and *H. vulgare*, before Cadenza, which ranked seventh. At both temperatures *B. distachyon* ranked the lowest for *V*_c_.

There is statistical evidence of a correlation between the performance of the genotypes with respect to *V*_c_ across temperature, with a Spearman’s rank correlation coefficient ρ=0.707 (*P*<0.001) (see Supplementary Tables S2 and S3). This value was slightly lower when comparing genotypes grouped according to *rbc*L sequence, although still significant (ρ=0.607, *P*=0.035) (Table 2).

**Table 2.** *Kinetic parameters of Rubisco at 25 and 35 °C according to the respective* rbc*L sequence (residues 1–463*) Where *n*>1, values are means±SEM for the Triticiae Rubiscos containing the same *rbc*L sequence. Other kinetic parameters are calculated using the Michaelis–Menten kinetic model as explained in text. For *S*_c/o_, *n*≥5 technical replicates per genotype.

Assay temp	Representative genotype	*n*	*K*_c_ (µM)	*V*_c_ (µmol min^–1^ mg^–1^)	*K*_o_ (µM)	*V*_o_ (µmol min^–1^ mg^–1^)	*S*_c/o_	*k*_cat_/*K*_c_ (21% O_2_)	Rank (*K*_c_)	Rank (*V*_c_)	Rank (*S*_c/o_)
25 °C	*T. aestivum* cv Cadenza	12	16.3±0.4	3.01±0.03	431.6±12.3	0.85±0.02	95.94±0.96	0.14±0.00	6	4	7
	*H. vulgare*	1	15.2±2.4	3.47±0.16	465.3±27.8	1.09±0.14	101.96±5.21	0.17	3	1	4
	*Aegilops* spp.	9	15.6±1.0	2.63±0.08	431.4±17.6	0.74±0.02	100.84±2.20	0.13±0.01	4	6	5
	*Ae. cylindrica*	1	13.7±2.9	3.2±0.20	451.0±11.4	0.97±0.02	108.9±3.80	0.17	2	2	2
	*Triticale* (Cando)	1	16.1±2.4	3.15±0.14	384.0±4.7	0.75±0.01	99.73±5.08	0.14	5	3	6
	*Ae. speltoides*	1	16.5±3.2	2.82±0.17	446.9±11.0	0.84±0.02	102.3±5.6	0.13	7	5	3
	*B. distachyon*	1	11.9±2.5	1.78±0.10	395.7±18.8	0.54±0.03	111±4.00	0.1	1	7	1
35 °C	*T. aestivum* cv Cadenza	12	28.5±1.6	6.55±0.20	363.2±7.1	1.07±0.03	79.69±2.38	0.18±0.01	7	2	6
	*H. vulgare*	1	24.4±3.2	6.69±0.34	315.2±15.6	0.97±0.05	89.4±4.16	0.2	6	1	2
	*Aegilops* spp.	9	23.0±0.8	5.1±0.21	382.1±10.6	1.01±0.05	87.09±1.23	0.17±0.01	4	5	5
	*Ae. cylindrica*	1	20.7±3.7	4.91±0.32	310.1±2.8	0.83±0.01	89.1±1.60	0.17	2	6	3
	Triticale (Cando)	1	21.5±3.7	5.9±0.37	360.1±26.0	1.30±0.09	76.14±2.76	0.2	3	3	7
	*Ae. speltoides*	1	23.9±4.0	5.66±0.35	382.1±21.7	1.02±0.06	88.5±2.2	0.18	5	4	4
	*B. distachyon*	1	18.1±2.5	3.62±0.17	431.7±27.3	0.92±0.06	94±1.80	0.16	1	7	1

Rubisco from 14 genotypes had a higher affinity for CO_2_ (lower *K*_c_) than Cadenza at 25 °C, with *B. distachyon* ranking the highest (see Supplementary Table S2). Similarly, at 35 °C 13 genotypes showed a higher affinity for CO_2_ (lower *K*_c_) compared with Cadenza (Supplementary Table S3). Of these, only Rubisco from *H. vulgare* showed both a higher *V*_c_ and a higher affinity for CO_2_ compared with Cadenza based on rank.

Regardless of the measurement temperature, Rubisco from most of the genotypes had a lower maximum oxygenation velocity (*V*_o_) than Cadenza. Rubisco from genotypes that had a low affinity for O_2_ (i.e. a high *K*_o_) at 25 °C also showed a relatively low affinity for O_2_ at 35 °C (Table 2 and Supplementary Tables S2 and S3).

**Fig. 2.**
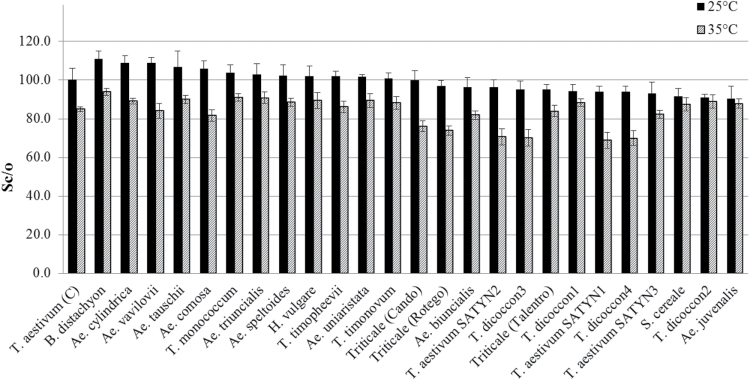
Specificity factor of Rubisco (*S*_c/o_) at 25 °C (black bars) and 35 °C (hatched bars) in 25 Triticeae genotypes. Data organized in decreasing rank at 25 °C, except for *T. aestivum* cv Cadenza, which is shown on the far left-hand side for comparison.

Rubisco specificity factor (*S*_c/o_) ranged from 90.4 (for *Ae. juvenalis*) to 111.0 (for *B. distachyon*) at 25 °C and was lower at 35 °C for all species (Fig. 2 and Supplementary Tables S2 and S3), ranging from 68.8 for *T. aestivum* SATYN1 to 94.0 for *B. distachyon*. In contrast to its ranking with respect to *V*_c_, *B. distachyon*, ranked the highest for *S*_c/o_ at both temperatures. The CIMMYT lines *T. aestivum* SATYN3, *T. dicoccon*1, and *T. dicoccon*2 ranked much higher at 35 °C than at 25 °C with respect to *S*_c/o_, although only *T. dicoccon*1 and *T. dicoccon*2 ranked higher than Cadenza. A correlation was identified in the performance of the genotypes across temperature with respect to *S*_c/o_ (ρ=0.857, *P*=0.003; Fig. 2 and Supplementary Tables S2 and S3). The correlation coefficient for *S*_c/o_ across temperature was lower but still significant when genotypes were grouped according to *rbc*L (ρ=0.503, *P*=0.002; Table 2). To compare *S*_c/o_ across temperatures, the respective ranks of individual genotypes were added up and compared (see Supplementary Tables S2 and S3). This revealed that *B. distachyon* (sum of ranks=2), *Ae. tauschii* (sum of ranks=8), *T. monococcum* (sum of ranks=8), *Ae. cylindrica* (sum of ranks=9) and *Ae. triuncialis* (sum of ranks=10) maintained their ranking much better than Cadenza (sum of ranks=28) across different temperatures.

**Fig. 3.**
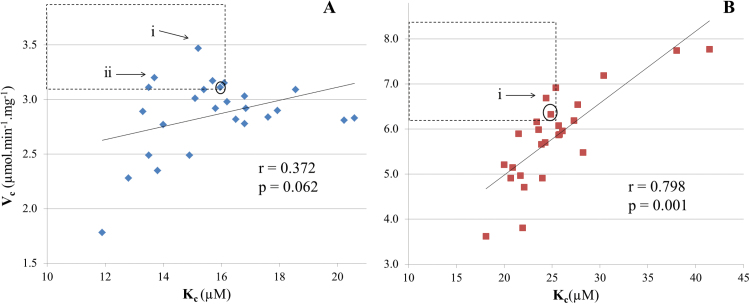
Relationship between *V*_c_ and *K*_c_ for Rubisco from 25 Triticeae genotypes at 25 °C (A) and 35 °C (B) in the absence of O_2_. Regression lines indicate the best fit through the data. Correlation coefficients (*r*) and *P*-values shown. The data point represented by *Triticum aestivum* cv Cadenza is highlighted by a circle. The area in the graph where Rubiscos with superior characteristics would be found is outlined. Arrows indicate genotypes with potentially superior Rubisco properties compared with Cadenza wheat. (i) *H. vulgare*; (ii) *Ae. cylindrica*.

A positive correlation was observed between Rubisco *V*_c_ and *K*_c_ for all 25 genotypes, with this correlation being stronger at 35 °C (*r*=0.798, *P*<0.001) than at 25 °C (*r*=0.372, *P*=0.062) (Fig. 3). Rubisco from two genotypes (*Ae. cylindrica* and *H. vulgare*) appeared to have superior catalytic properties at 25 °C, possessing higher *V*_c_ and lower *K*_c_ values than Cadenza. From these, only Rubisco from *H. vulgare* retained superior properties at 35 °C compared with Cadenza, which performed remarkably well at this higher temperature. A strong positive correlation was found between *V*_c_ and *V*_o_ at 25 °C (*r*=0.726, *P*<0.001), while a moderate negative correlation was found between *V*_c_ and *S*_c/o_ at both temperatures (*r*=–0.428, *P*=0.029 at 25 °C and *r*=–0.528, *P*=0.006 at 35 °C).

Analysis of the *rbc*L coding sequences (codons 1–463) of the 25 genotypes revealed differences relative to the Cadenza reference sequence in 12 corresponding large subunit (LSu) residues, at positions spanning a number of domains within the Rubisco large subunit structure (Table 3). For 11 genotypes, all of which were either *Triticum* or Triticale, LSu sequences were identical to Cadenza at the amino acid level (Table 3 and Supplementary Table S1). Of the remaining 14 genotypes, each possessed at least one different amino acid from the Cadenza LSu. The LSu residue differences K14Q and S95N were the most common, and were found to occur together in all but one *Aegilops* species. These two residue differences were also found in *S. cereale* and *T. monococcum*. In *H. vulgare*, K14Q was the only difference relative to Cadenza *rbc*L. In *Ae. cylindrica*, in addition to K14Q and S95N, the LSu sequence also contained the difference V17A compared with Cadenza.

**Table 3.** *Amino acid differences in the Rubisco large subunit predicted protein sequences for 25 Triticeae genotypes relative to* T. aestivum *cv Cadenza* Residues under positive selection (Kapralov and Filatov, 2007, Galmés *et al.* 2014b) are indicated with an asterisk. Functional interactions described in the literature for these residues as indicated (AS, active site; ID, intradimer interactions; DD, dimer:dimer interactions; RA, interactions with Rubisco activase; SSU, interaction with small subunits). Symbols and colours match those used in Fig. 4. na, not applicable.

Residue change	Symbol	Interaction	Location of residue	Species
na	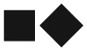			*T. aestivum* cv. Cadenza
na	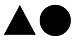			*T. aestivum* SATYN1
				*T. aestivum* SATYN2
				*T. aestivum* SATYN3
				*T. dicoccon*1
				*T. dicoccon*2
				*T. dicoccon*3
				*T. dicoccon*4
				*T. timonovum*
				*T. timopheevii*
				*Triticale* (Talentro)
				*Triticale* (Rotego)
K14Q*	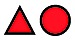		N-terminal	*H. vulgare* cv. Lenins
K14Q*	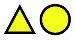		N-terminal	*Ae. tauschii*
S95N*		ID, RA		*Ae. juvenalis*
				*Ae. vavilovii*
				*Ae. biuncialis*
				*Ae. triuncialis*
				*Ae. comosa*
				*Ae. uniaristata*
				*S. cereale* cv. Agronom
				*T. monococcum*
K14Q*	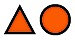		N-terminal	*Ae. cylindrica*
V17A			N-terminal	
S95N*				
G47W	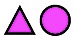	ID	Strand B	*Triticale* (Cando)
K81R	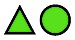			*Ae. speltoides*
I225T*		SSU	Helix 2	
G10S	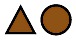		N-terminal	*B. distachyon*
K21R			N-terminal	
A91P*		RA		
I251M*		DD, ID, SSU	Helix 3	
S328A*		AS	Loop 6	
M341I		AS, ID	Helix 6	

The catalytic efficiency of Rubisco in air (21% O_2_) can be measured as the ratio between the carboxylase turnover number and the Michaelis–Menten constant for CO_2_ (i.e. *k*_cat_/*K*_c_ at 21% O_2_, Table 2). Rubisco from *Ae. cylindrica* and *H. vulgare* appeared to have a superior efficiency to Cadenza (and other genotypes with the reference LSu sequence) at 25 °C. Rubisco from *H. vulgare* also showed superior efficiency at 35 °C, along with Triticale (Cando).

**Fig. 4.**
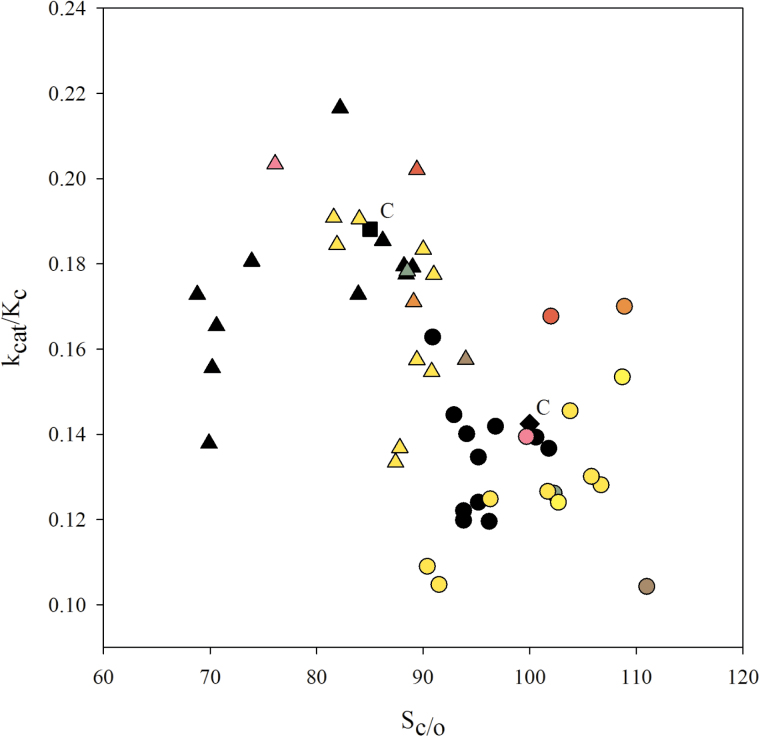
The relationship between the catalytic efficiency of Rubisco at 21% O_2_ (*k*_cat_/*K*_c_, µM s^−1^) and the specificity factor (*S*_c/o_) of Rubisco at 25 °C (circles) and 35 °C (triangles). Each colour denotes an *rbc*L sequence (as per Table 3) and Cadenza wheat (C, used as reference) is represented by the diamond and square at 25 and 35 °C, respectively.

From the relationship between catalytic efficiency (*k*_cat_/*K*_c_ at 21% O_2_) and *S*_c/o_ it is also possible to identify Rubisco enzymes with superior catalytic performance (Fig. 4). In general, catalytic efficiency was higher, but *S*_c/o_ was lower, at 35 °C compared with 25 °C. Forms of Rubisco with the same LSu sequence (residues 1–463) showed considerable variation in their combination of catalytic properties. Rubisco from *H. vulgare*, which only differs from Cadenza by virtue of the alternative residue Q14 (K14 in Cadenza), stood out as having a promising combination of *k*_cat_/*K*_c_ and *S*_c/o_ at both temperatures. Rubisco from *Ae. cylindrica*, differing by K14Q, V17A and S95N relative to Cadenza, showed promise only at 25 °C. Rubisco from *Ae. vavilovii* showed a similar catalytic response, despite the LSu sequence being identical to that from a number of other species that did not show such catalytic advantage compared with Cadenza. In *B. distachyon*, six residue changes compared with Cadenza (Table 3) might be associated with a higher *S*_c/o_, but at the expense of catalytic efficiency (Fig. 4).

**Fig. 5.**
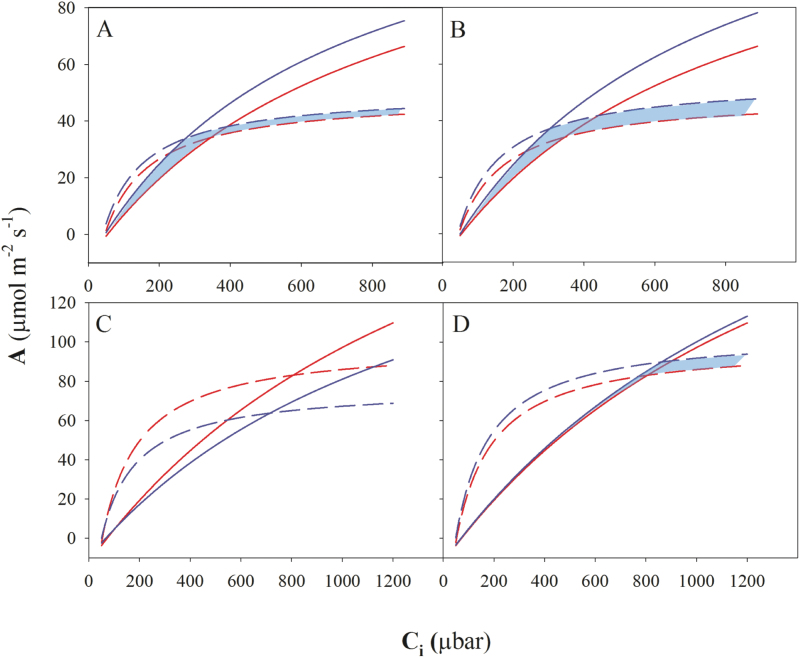
Modelling photosynthesis at 25 °C (A, B) and 35 °C (C, D), to demonstrate the benefit of replacing Rubisco of *T. aestivum* cv Cadenza (red) with Rubisco from *Ae. cylindrica* (A, C; blue) or *H. vulgare* (B, D; blue). Rubisco-limited (*A*_c_, solid lines) and RuBP regeneration-limited (*A*_j_, dashed lines) rates of net CO_2_ assimilation (*A*) were derived using the model of Farquhar et al. (1980) and the Rubisco catalytic constants measured *in vitro* for each genotype. Blue shading indicates where Rubisco from the test genotypes showed higher assimilation rates than native Cadenza Rubisco.

The Rubisco catalytic constants measured *in vitro* at 25 and 35 °C for Cadenza, *Ae. cylindrica* and *H. vulgare* were used to assess the theoretical impact on photosynthetic performance of wheat leaves over a range of intercellular CO_2_ concentrations, including those corresponding to ambient air (*c.* 210 µbar). This modelling exercise predicted that at 25 °C photosynthetic rates would be improved by replacing wheat Rubisco with the enzyme from either *Ae. cylindrica* or *H. vulgare* (Fig. 5A, B). At 25 °C Rubisco from *Ae. cylindrica* showed a maximal increase in assimilation rate of 23% (6.3 µmol m^−2^ s^−1^) compared with Cadenza at 270 μbar *C*_i_ (Fig. 5A), while Rubisco from *H. vulgare* maximally increased assimilation rate by 22% (6.7 µmol m^−2^ s^−1^) at 300 μbar *C*_i_ (Fig. 5B). Rubisco from *H. vulgare* also showed promise for the improvement of photosynthesis in wheat at 35 °C, while the enzyme from *Ae. cylindrica* was inferior to Cadenza wheat at the higher temperature (Fig. 5C, D).

## Discussion

The catalytic properties and primary sequence of the Rubisco large subunits (LSu, encoded by *rbc*L) from 25 Triticeae genotypes revealed diversity relevant to improving wheat photosynthetic performance in current and projected warmer temperatures. In the major wheat producing countries, grain filling is accompanied by increasing daytime temperatures (Asseng *et al.*, 2015). Within the limits of resources available to this study, measurements were taken at an ideal growth temperature (25 °C, Nagai and Makino, 2009), and at an elevated temperature at which a pronounced negative impact on yield would be expected (35 °C, Duncan *et al.*, 2014). At the higher temperature *V*_c_ was higher, but *S*_c/o_ was lower, which is consistent with previous research (Brooks and Farquhar, 1985; Tcherkez *et al.*, 2006; Savir *et al.*, 2010; Galmés *et al.*, 2014b). Differences were observed in the Rubisco response to temperature that suggests some acclimation to different geographical locations.

As reported previously (Savir *et al.*, 2010), a positive correlation between *V*_c_ and *K*_c_, the determinants of Rubisco carboxylase efficiency, was observed at both temperatures for the Triticeae genotypes studied here, indicating that genotypes with a high *V*_c_ tend to have lower affinity for CO_2_. The catalytic efficiency of Rubisco in air was a useful tool to identify Rubiscos with superior performance. Furthermore, the combined results suggest that all of the Rubisco catalytic properties, including the specificity factor, must be taken into account during the search for forms of Rubisco with improved performance in air. This follows from the parameters required for biochemical modelling of photosynthetic performance, which include *V*_c_, *K*_c_, and *S*_c/o_ (=*V*_c_.*K*_o_/*V*_o_.*K*_c_), the latter being used to determine the compensation point (Γ*=0.5[O_2_]/*S*_c/o_) in the absence of dark respiration (von Caemmerer, 2000).

In wheat, variation in *V*_c_ has been observed across different genotypes and it has been suggested that many of the catalytic properties of Rubisco are determined by the large subunit (Evans and Austin, 1986; Terachi *et al.*, 1987; Kasai *et al.*, 1997), which contains the catalytic sites. The *rbc*L gene is chloroplast encoded (Spreitzer and Salvucci, 2002) and the chloroplast genome tends to be evolutionarily highly conserved. However, within the Poaceae, *rbc*L has evolved at a relatively rapid rate compared with other families of flowering plants (Bousquet *et al.*, 1992; Gaut *et al.*, 1992). Since the large subunits contribute directly to catalytic function, variation in this sequence in wheat relatives represents a potential source of improved catalytic activity.

The majority of the Triticeae genotypes characterized in this study are highly inter-related and this was reflected in the similarity of the respective *rbc*L sequences. Some of the observed differences in Rubisco catalytic activity correlated with differences in *rbc*L sequence. For example, differences in catalytic properties determined for *H. vulgare*, *Ae. cylindrica*, Triticale (Cando) and *B. distachyon* compared with Cadenza Rubisco might be associated with their specific *rbc*L sequences. Conversely, differences in catalysis for genotypes with the same *rbc*L sequence (e.g. the Cadenza group represented by black symbols or the *Aegilops* group represented by yellow symbols in Fig. 4) may be associated with either changes in the extreme C-terminus whose sequence was not determined or, more likely, diversity in the small subunit sequence (Guo *et al.*, 1997; Spreitzer, 2003; Spreitzer *et al.*, 2005; Ishikawa *et al.*, 2011; Cai *et al.*, 2014; Morita *et al.*, 2014). Future studies to characterize the exact number and relative expression of small subunit genes in wheat and wheat relatives may reveal novel avenues for improving Rubisco catalysis and photosynthesis.

When comparing kinetic parameters between species grouped by LSu sequence (Table 2), *H. vulgare* (difference K14Q) ranked highest in *V*_c_ at both 25 and 35 °C, with only *Ae. cylindrica* (K14Q, V17A, and S95N) also ranking higher than species with the control sequence at 25 °C. Rubisco from *Ae. cylindrica* also had a lower *K*_c_ (higher affinity for CO_2_) at both temperatures compared with Rubisco from species with the reference *rbc*L sequence.

When present as lysine, large subunit residue 14 is known to be a site of post-translational tri-methylation in many flowering plant species, but not *T. aestivum* (Houtz *et al.*, 2008). Available data show that glutamine is the only alternative residue found at this position (K14Q, Houtz *et al.*, 1989; Houtz *et al.*, 1992; Trievel *et al.*, 2003), although the importance of this position and its modification remains unresolved (Houtz *et al.*, 2008). The results presented here suggest the possibility that either the amino acid difference itself (K14Q) or the absence of methylation at this position alters Rubisco kinetics in a manner favourable to photosynthesis.

The other residue difference common to most of the *Aegilops*, *S. cereale* and *T. monococum*, S95N, occurs in a poorly conserved region of the *rbc*L gene, which is in the proximity of residues known to be involved in interactions with Rubisco activase (Portis, 2003; Portis *et al.*, 2008; Carmo-Silva *et al.*, 2014). Recently, this residue was highlighted during a search for residues under positive selection, and it is in proximity to residues involved in L_2_ intradimer interactions (Galmés *et al.*, 2014b). Interestingly, species combining the K14Q and S95N residue differences (but having no other differences) showed no consistent catalytic difference compared with the reference LSu sequence group.

The valine at position 17 is known to be involved in intradimer interactions (Knight *et al.*, 1990; Kellogg and Juliano, 1997). Rubisco from *Ae. cylindrica* containing V17A in combination with K14Q and S95N had improved carboxylation catalytic efficiency at 21% O_2_ and 25 °C compared with the reference Cadenza (Table 2). This form of Rubisco was predicted by modelling to improve photosynthetic performance at all CO_2_ levels relative to Cadenza at 25 °C (Fig. 5A). Hence, the influence exerted by the relatively conservative Val–Ala (V17A) difference, when combined with K14Q (and S95N), may explain the positive impact on Rubisco catalysis.

*H. vulgare* Rubisco had improved *k*_cat_/*K*_c_ compared with Cadenza, while only containing the K14Q difference. Studies in *Anacystis nidulans* found that a K14Q or K14L mutation had no influence on enzyme activity (Kettleborough *et al.*, 1991). While the present study did not cover the extreme C-terminus of the large subunit, available sequences showed a KV extension in that region of the *H. vulgare* sequence (Petersen and Seberg, 2003), which may be relevant to its superior catalysis in comparison to Cadenza. Confirmation of this hypothesis would be valuable, given that modelling of the photosynthetic response to intercellular CO_2_ predicts a benefit at both 25 and 35 °C by replacing the native Rubisco with the barley enzyme.

As with *Ae. cylindrica* (Colmer *et al.*, 2006), barley is considered to be a valuable genetic resource for improving stress tolerance in wheat (Dulai *et al.*, 2011; Molnar-Lang *et al.*, 2014). Barley–wheat hybrids have been investigated before (Kruse, 1973; Malik *et al.*, 2011; Dulai *et al.*, 2011; Rodríguez-Suárez *et al.*, 2011; Pershina *et al.*, 2012; Zou *et al.*, 2012), but without a focus on yield improvement. One notable exception is the development of Tritordeum, which is a hybrid between wild barley (*H. chilense*) and durum wheat (*T. turgidum ssp. Durum*, haploid genome BA; Martin *et al.* 1996), which has been commercialized (http://www.agrasys.es/). Tritordeum has particularly high protein content and has shown tolerance to drought conditions in field trials (Martin *et al.*, 1999; Villegas *et al.*, 2010). While Tritordeum does not include the D genome present in *T. aestivum*, data in this study suggest that this hybrid warrants further investigation with respect to Rubisco kinetics and yield potential.

This study has identified residues that warrant further study, e.g. by mutagenesis. Well targeted single amino acid changes can have a dramatic impact on catalytic performance (e.g. Whitney *et al.*, 2011). However, at present there is no available expression system to test the effect of amino acid substitutions on Rubisco from monocots. An alternative, and possibly more promising approach, which utilizes available technology, is the introgression of traits through wide-crossing of Triticeae genotypes.

## Conclusion

The Rubisco catalytic properties determined for 25 genotypes showed that variation exists even amongst closely related genotypes. Rubisco from *Ae. cylindrica* and *H. vulgare* showed promising catalytic properties that should be explored in the context of improving photosynthesis, and ultimately yield, in wheat. Ideally, this could be carried out by crossing a number of the species examined here with bread wheat and studying the resulting plants with respect to Rubisco catalytic activity, photosynthesis and yield. This study supports the case for investment in genetic resource screening for photosynthesis-related characteristics.

## Supplementary data

Supplementary data are available at *JXB* online.

Table S1. Rubisco large subunit (*rbc*L) single nucleotide polymorphisms.

Table S2. Rubisco catalytic parameters at 25 °C.

Table S3. Rubisco catalytic parameters at 35 °C.

Supplementary Data
