# Supplementary material for: Rubisco catalytic properties of wild and domesticated relatives provide scope for improving wheat photosynthesis
Source: J Exp Bot. 2016 Jan 21;67(6):1827–38. doi: 10.1093/jxb/erv574 (PMC4783365; doi:10.1093/jxb/erv574)
Supplement: Supplementary Data [file supp_67_6_1827__index.html]

Rubisco catalytic properties of wild and domesticated relatives provide scope for improving wheat photosynthesis — Rubisco catalytic properties of wild and domesticated relatives provide scope for improving wheat photosynthesis — Rubisco catalytic properties of wild and domesticated relatives provide scope for improving wheat photosynthesis — Supplementary Data 

# Rubisco catalytic properties of wild and domesticated relatives provide scope for improving wheat photosynthesis

## Supplementary Data

Data files

- supplementary\_tables\_S1\_S3.pdf - Supplementary Data
