# Supplementary material for: Rubisco catalytic properties of wild and domesticated relatives provide scope for improving wheat photosynthesis
Source: J Exp Bot. 2016 Jan 21;67(6):1827–38. doi: 10.1093/jxb/erv574 (PMC4783365; doi:10.1093/jxb/erv574)
Supplement: Supplementary Data [file supp_erv574_supplementary_tables_S1_S3.pdf]

Supporting Information

Article title: **Rubisco catalytic properties of wild and domesticated relatives provide scope for improving wheat photosynthesis**

Authors: Anneke Prins, Douglas J. Orr, P. John Andralojc, Matthew P. Reynolds, Elizabete Carmo-Silva, Martin A. J. Parry

The following Supporting Information is available for this article:

Table S1 Rubisco large subunit (*rbc*L) single nucleotide polymorphisms.

Table S2 Rubisco catalytic parameters at 25°C.

Table S3 Rubisco catalytic parameters at 35°C.

Table S1. Summary of Rubisco large subunit (*rbc*L) single nucleotide polymorphisms and non-synonymous codon differences between *Triticum aestivum* cv Cadenza and 25 Triticeae genotypes. For each genotype, 1389 nucleotides of *rbc*L coding sequence were determined (corresponding to residues 1-463, i.e. 98% of the gene). Differences in nucleotides and corresponding amino acids are shown, as well as the accession numbers for each sequence (EMBL; http://www.ebi.ac.uk/ena/).

Species	SNPs	Synonymous (silent)	Residue details			EMBL Accession
			Residue	*T. aestivum* cv. Cadenza	Substitute	
T. aestivum cv. Cadenza						LN626616
T. aestivum SATYN1	3	3	-	-	-	LN626617
T. aestivum SATYN2	4	4	-	-	-	LN626618
T. aestivum SATYN3	3	3	-	-	-	LN626619
T. monococcum	6	4	14	Lys (AAA)	Gln (CAA)	LN626620
			95	Ser (AGC)	Asn (AAC)	
T. timopheevii	3	3	-	-	-	LN626621
T. timonovum	4	4	-	-	-	LN626622
*T. dicoccon*1	4	4	-	-	-	LN626623
*T. dicoccon*2	3	3	-	-	-	LN626624
*T. dicoccon*3	3	3	-	-	-	LN626625
*T. dicoccon*4	3	3	-	-	-	LN626626
Ae. cylindrica	6	3	14	Lys (AAA)	Gln (CAA)	LN626627
			17	Val (GTT)	Ala (GCT)	
			95	Ser (AGC)	Asn (AAC)	
Ae. juvenalis	5	3	14	Lys (AAA)	Gln (CAA)	LN626628
			95	Ser (AGC)	Asn (AAC)	
Ae. speltoides	7	4	81	Lys (AAA)	Arg (AGA)	LN626629
			225	Ile (ATT)	Thr (ACC)	
Ae. tauschii	6	4	14	Lys (AAA)	Gln (CAA)	LN626630
			95	Ser (AGC)	Asn (AAC)	
Ae. uniaristata	5	3	14	Lys (AAA)	Gln (CAA)	LN626631
			95	Ser (AGC)	Asn (AAC)	
Ae. comosa	5	3	14	Lys (AAA)	Gln (CAA)	LN626632
			95	Ser (AGC)	Asn (AAC)	
Ae. biuncialis	5	3	14	Lys (AAA)	Gln (CAA)	LN626633
			95	Ser (AGC)	Asn (AAC)	
Ae. triuncialis	5	3	14	Lys (AAA)	Gln (CAA)	LN626634
			95	Ser (AGC)	Asn (AAC)	
Ae. vavilovii	5	3	14	Lys (AAA)	Gln (CAA)	LN626635
			95	Ser (AGC)	Asn (AAC)	
Triticale (Talentro)	4	4	-	-	-	LN626636
Triticale (Rotego)	3	3	-	-	-	LN626637
Triticale (Cando)	5	4	47	Gly (GGG)	Trp (TGG)	LN626638
S. cereale cv. Agronom	6	4	14	Lys (AAA)	Gln (CAA)	LN626639
			95	Ser (AGC)	Asn (AAC)	
B. distachyon	34	28	10	Gly (GGT)	Ser (AGT)	LN626640
			21	Lys (AAA)	Arg (AGA)	
			91	Ala (GCT)	Pro (CCT)	
			251	Ile (ATT)	Met (ATG)	
			328	Ser (TCC)	Ala (GCC)	
			341	Met (ATG)	Ile (ATC)	
H. vulgare cv. Lenins	15	14	14	Lys (AAA)	Gln (CAA)	LN626641

Table S2. Rubisco catalytic parameters measured at 25°C for 25 different Triticeae genotypes and *T. aestivum* cv Cadenza (C), used as control. Genotypes grouped according to *rbc*L sequence (see Table 3 in main text). Specificity ($S_{c/o}$) values are mean ± SE (n≥5). All other catalytic values are calculated using the Michaelis Menten kinetic model (see main text). V_c, V_o – maximal enzyme velocity for carboxylation and oxygenation. K_c, K_o – Michaelis Menten constant for carboxylation and oxygenation. k_{cat} – turnover number. k_{cat}/K_c – carboxylation efficiency. The highest value is ranked as 1.

Species name	V_c (μmol min^{-1} mg^{-1})	K_c (μM)	k_{cat} CO_2	V_o (μmol min^{-1} mg^{-1})	K_o (μM)	k_{cat} O_2	$S_{c/o}$	k_{cat}/K_c (21% O_2)	Rank (V_c)	Rank (K_c)	Rank ($S_{c/o}$)
T. aestivum (C)	3.11 ± 0.07	16.0 ± 1.2	3.57	0.81 ± 0.02	401.5 ± 8.4	0.93	100.0 ± 6.0	0.14	5.5	15	13
T. aestivum SATYN1	2.84 ± 0.14	17.6 ± 2.5	3.26	0.81 ± 0.02	469.1 ± 11.5	0.93	93.8 ± 3.2	0.12	16	22	21.5
T. aestivum SATYN2	2.90 ± 0.16	17.9 ± 2.8	3.33	0.78 ± 0.02	465.4 ± 11.3	0.90	96.2 ± 3.8	0.12	14	23	17
T. aestivum SATYN3	3.17 ± 0.14	15.7 ± 2.2	3.64	0.82 ± 0.03	379.0 ± 13.5	0.95	92.9 ± 5.9	0.14	3	13	23
*T. dicoccon*1	3.09 ± 0.13	15.4 ± 2.1	3.55	0.85 ± 0.02	398.7 ± 10.5	0.98	94.1 ± 3.8	0.14	7.5	12	20
*T. dicoccon*2	3.11 ± 0.15	13.5 ± 2.3	3.57	1.01 ± 0.06	397.0 ± 21.9	1.16	90.9 ± 1.7	0.16	5.5	4.5	25
*T. dicoccon*3	2.92 ± 0.15	16.8 ± 2.5	3.36	0.83 ± 0.07	455.5 ± 37.3	0.95	95.2 ± 4.2	0.12	12.5	20	18.5
*T. dicoccon*4	3.09 ± 0.16	18.6 ± 2.7	3.55	0.86 ± 0.09	484.6 ± 48.8	0.99	93.8 ± 3.2	0.12	7.5	24	21.5
T. timonovum	3.03 ± 0.18	16.8 ± 3.3	3.48	0.99 ± 0.07	495.1 ± 34.7	1.14	100.6 ± 3.2	0.14	9	20	12
T. timopheevii	2.98 ± 0.13	16.2 ± 2.3	3.43	0.78 ± 0.01	429.0 ± 4.6	0.89	101.8 ± 2.8	0.14	11	17	10
Triticale (Talentro)	2.92 ± 0.15	15.8 ± 2.8	3.36	0.89 ± 0.01	435.5 ± 6.7	1.02	95.2 ± 2.6	0.13	12.5	14	18.5
Triticale (Rotego)	3.01 ± 0.14	15.1 ± 2.4	3.46	0.76 ± 0.02	368.7 ± 11.5	0.87	96.8 ± 3.1	0.14	10	10	15
H. vulgare cv. Lenins	3.47 ± 0.16	15.2 ± 2.4	3.99	0.81 ± 0.02	465.3 ± 27.8	1.20	102.0 ± 5.2	0.17	1	11	9
Ae. tauschii	2.49 ± 0.09	14.9 ± 1.8	2.86	0.81 ± 0.02	495.0 ± 27.5	0.89	106.7 ± 8.3	0.13	22.5	9	4
Ae. juvenalis	2.83 ± 0.18	20.6 ± 3.8	3.25	0.78 ± 0.02	492.0 ± 21.5	0.86	90.4 ± 6.4	0.11	17	26	26
Ae. vavilovii	2.89 ± 0.12	13.3 ± 1.9	3.32	0.82 ± 0.03	362.6 ± 26.9	0.83	108.7 ± 3.0	0.15	15	3	3
Ae. biuncialis	2.78 ± 0.15	16.8 ± 2.9	3.2	0.85 ± 0.02	469.9 ± 15.1	0.93	96.3 ± 4.9	0.12	20	20	16
Ae. triuncialis	2.28 ± 0.11	12.8 ± 2.2	2.62	1.01 ± 0.06	380.0 ± 8.8	0.76	102.7 ± 5.9	0.12	25	2	7
Ae. comosa	2.49 ± 0.11	13.5 ± 2.1	2.86	0.83 ± 0.07	359.5 ± 9.0	0.73	105.8 ± 4.3	0.13	22.5	4.5	5
Ae. uniaristata	2.35 ± 0.11	13.8 ± 2.3	2.7	0.86 ± 0.09	450.5 ± 18.1	0.98	101.7 ± 1.1	0.13	24	7	11
S. cereale cv. Agronom	2.81 ± 0.10	20.2 ± 2.1	3.23	0.99 ± 0.07	472.4 ± 12.9	0.82	91.5 ± 4.1	0.1	19	25	24
T. monococcum	2.77 ± 0.12	14.0 ± 2.1	3.18	0.78 ± 0.01	401.0 ± 21.1	0.88	103.8 ± 4.1	0.15	21	8	6
Ae. cylindrica	3.20 ± 0.20	13.7 ± 2.9	3.68	0.97 ± 0.02	451.0 ± 11.4	1.11	108.9 ± 3.8	0.17	2	6	2
Triticale (Cando)	3.15 ± 0.14	16.1 ± 2.4	3.62	0.75 ± 0.01	384.0 ± 4.7	0.87	99.7 ± 5.1	0.14	4	16	14
Ae. speltoides	2.82 ± 0.17	16.5 ± 3.2	3.24	0.84 ± 0.02	446.9 ± 11.0	0.97	102.3 ± 5.6	0.13	18	18	8
B. distachyon	1.78 ± 0.10	11.9 ± 2.5	2.05	0.54 ± 0.03	395.7 ± 18.8	0.62	111.0 ± 4.0	0.1	26	1	1

Table S3. Rubisco catalytic parameters measured at 35°C for 25 different Triticeae genotypes and *T. aestivum* cv Cadenza (C), used as control. Genotypes grouped according to *rbc*L sequence (see Table 3 in main text). Specificity ($S_{c/o}$) values are mean ± SE (n≥5). All other catalytic values are calculated using the Michaelis Menten kinetic model (see main text). V_c, V_o – maximal enzyme velocity for carboxylation and oxygenation. K_c, K_o – Michaelis Menten constant for carboxylation and oxygenation. k_{cat} – turnover number. k_{cat}/K_c – carboxylation efficiency. The highest value is ranked as 1.

Species name	V_c (µmol min^{-1} mg^{-1})	K_c (µM)	k_{cat} CO_2	V_o (µmol min^{-1} mg^{-1})	K_o (µM)	k_{cat} O_2	$S_{c/o}$	k_{cat}/K_c (21% O_2)	Rank (V_c)	Rank (K_c)	Rank ($S_{c/o}$)
T. aestivum (C)	6.33 ± 0.16	24.9 ± 1.6	7.27	1.13 ± 0.07	372.6 ± 17.0	1.30	85.0 ± 1.2	0.19	7	15	15
T. aestivum SATYN1	5.87 ± 0.20	25.7 ± 2.1	6.75	1.24 ± 0.40	373.1 ± 120.6	1.43	68.8 ± 4.3	0.17	15	17.5	26
T. aestivum SATYN2	6.19 ± 0.25	27.3 ± 2.6	7.11	1.26 ± 0.14	391.6 ± 43.7	1.45	70.6 ± 4.1	0.17	8	21	23
T. aestivum SATYN3	6.92 ± 0.35	25.4 ± 3.2	7.95	1.10 ± 0.06	331.3 ± 19.2	1.26	82.2 ± 2.1	0.22	4	16	18
*T. dicoccon*1	6.54 ± 0.36	27.7 ± 3.8	7.52	0.94 ± 0.06	351.2 ± 22.7	1.08	88.4 ± 2.0	0.18	6	22	10
*T. dicoccon*2	7.19 ± 0.30	30.4 ± 3.0	8.26	0.95 ± 0.04	355.9 ± 15.4	1.09	89.0 ± 3.5	0.18	3	24	8
*T. dicoccon*3	7.74 ± 0.40	38.0 ± 4.4	8.9	1.14 ± 0.02	392.1 ± 8.6	1.31	70.2 ± 4.3	0.16	2	25	24
*T. dicoccon*4	7.77 ± 0.44	41.4 ± 5.0	8.93	0.97 ± 0.12	360.0 ± 44.0	1.11	69.9 ± 3.9	0.14	1	26	25
T. timonovum	6.08 ± 0.28	25.7 ± 3.0	6.99	1.02 ± 0.08	382.0 ± 28.2	1.18	88.2 ± 3.4	0.18	10	17.5	11
T. timopheevii	5.88 ± 0.24	25.8 ± 2.6	6.76	0.92 ± 0.12	349.1 ± 44.6	1.06	86.2 ± 3.0	0.19	14	19	14
Triticale (Talentro)	5.96 ± 0.32	26.1 ± 3.6	6.85	1.05 ± 0.13	386.0 ± 47.0	1.21	83.9 ± 3.0	0.17	12	20	17
Triticale (Rotego)	6.16 ± 0.32	23.4 ± 3.2	7.08	1.12 ± 0.11	313.4 ± 31.9	1.28	73.9 ± 2.4	0.18	9	9	22
H. vulgare cv. Lenins	6.69 ± 0.34	24.4 ± 3.2	7.69	1.13 ± 0.07	315.2 ± 15.6	1.11	89.4 ± 4.2	0.2	5	14	5.5
Ae. tauschii	4.97 ± 0.27	21.7 ± 3.1	5.71	1.24 ± 0.40	398.3 ± 47.9	1.16	90.0 ± 2.1	0.18	21	6	4
Ae. juvenalis	3.81 ± 0.16	21.9 ± 2.5	4.37	1.26 ± 0.14	421.1 ± 43.2	1.21	87.8 ± 2.4	0.14	25	7	12
Ae. vavilovii	5.99 ± 0.27	23.6 ± 2.8	6.89	1.10 ± 0.06	365.0 ± 24.4	1.27	84.0 ± 3.8	0.19	11	10	16
Ae. biuncialis	5.15 ± 0.22	20.9 ± 2.4	5.92	0.94 ± 0.06	392.1 ± 4.3	1.36	81.9 ± 2.3	0.18	20	4	19
Ae. triuncialis	4.91 ± 0.21	24.0 ± 2.6	5.64	0.95 ± 0.04	339.4 ± 34.1	0.88	90.8 ± 2.9	0.15	22.5	12	3
Ae. comosa	5.21 ± 0.31	20.0 ± 3.4	5.99	1.14 ± 0.02	403.0 ± 14.1	1.48	81.6 ± 2.9	0.19	19	2	20
Ae. uniaristata	4.71 ± 0.23	22.1 ± 3.0	5.41	0.97 ± 0.12	343.2 ± 52.9	0.94	89.4 ± 3.7	0.16	24	8	5.5
S. cereale cv. Agronom	5.48 ± 0.27	28.2 ± 3.5	6.3	1.02 ± 0.08	427.3 ± 6.6	1.09	87.4 ± 3.5	0.13	18	23	13
T. monococcum	5.70 ± 0.27	24.3 ± 3.0	6.55	0.92 ± 0.12	349.5 ± 22.2	1.04	91.0 ± 2.0	0.18	16	13	2
Ae. cylindrica	4.91 ± 0.32	20.7 ± 3.7	5.64	0.83 ± 0.01	310.1 ± 2.8	0.95	89.1 ± 1.6	0.17	22.5	3	7
Triticale (Cando)	5.90 ± 0.37	21.5 ± 3.7	6.78	1.30 ± 0.09	360.1 ± 26.0	1.49	76.1 ± 2.8	0.2	13	5	21
Ae. speltoides	5.66 ± 0.35	23.9 ± 4.0	6.51	1.02 ± 0.06	382.1 ± 21.7	1.18	88.5 ± 2.2	0.18	17	11	9
B. distachyon	3.62 ± 0.17	18.1 ± 2.5	4.16	0.92 ± 0.06	431.7 ± 27.3	1.06	94.0 ± 1.8	0.16	26	1	1
